# DYNAMICS IN INTERGENERATIONAL FAMILY RELATIONSHIPS IN THE CHINESE CONTEXT

**DOI:** 10.1093/geroni/igae098.1600

**Published:** 2024-12-31

**Authors:** Tianyuan Li, Helene H Fung, Christiane Hoppmann

**Affiliations:** Chair; Co-Chair; Discussant

## Abstract

Various intergenerational family relationships play essential roles in maintaining older adults’ well-being and promoting successful aging. The intergenerational family relationships are particularly important in the Chinese culture as it has a long tradition emphasizing familism values. This symposium consists of four studies which investigate the dynamics of intergenerational family relationships in the Chinese context at different stages of later adulthood. The intergenerational relationships covered range from the relationship between aging parents and their adult children (Jiang et al.), grandparents providing care to young grandchildren (Hu et al.), the relationship between grandparents and adult grandchildren (Li et al.), and grandparents receiving care from family members (You et al.). The results jointly support the importance of the bidirectional support between the older and younger generations and demonstrate how the balance of support evolves over time. Chinese Gerontology Studies Interest Group Sponsored Symposium

